# Supplementary material for: The genome sequence of the Broad Centurion soldierfly, Chloromyia formosa (Scopoli, 1763)
Source: Wellcome Open Res. 2025 May 23;10:266. [Version 1] doi: 10.12688/wellcomeopenres.24255.1 (PMC12187159; doi:10.12688/wellcomeopenres.24255.1)
Supplement: Peer review history

# Reviewer report: Reviewer response for version 1

**DOI:** 10.21956/wellcomeopenres.26752.r124267

**Published:** 2025-06-25

**Authors:** Terrence Sylvester

**Affiliations:** 1 University of memphis, memphis, USA

The authors provide a high-quality genome assembly of the Broad Centurion soldierfly, *Chloromyia formosa*.

 Authors provide detailed background information on its morphology, life history and geographic distribution.

 The authors conducted a preliminary genome size estimation using k-mer-based analysis of the HiFi reads, which served as a benchmark to assess the completeness and plausibility of the final assembly. While the final assembly size is somewhat larger than the estimated genome size, this discrepancy is not unusual and is likely due to the inherent limitations of k-mer-based estimators, which can both over- and underestimate genome size depending on heterozygosity, repeat content, and sequencing depth.

 The resulting genome assembly appears to be of high quality, supported by a range of standard genome quality metrics (e.g., contiguity, completeness, and error correction). The authors also note that the assembly was curated to minimise structural and base-level errors, which further increases its reliability as a reference genome.

 This genome will likely serve as a valuable resource for the broader research community, facilitating studies in comparative genomics, functional genomics, and evolutionary biology. To further enhance the utility of this resource, I recommend that the authors include additional information on the quality and characteristics of the DNA used for PacBio sequencing. Specifically, the inclusion of quality control metrics such as Nanodrop spectra, Femto Pulse or Bioanalyzer fragment size distributions, and fluorometric quantifications (e.g., Qubit) would be highly beneficial. These data would provide researchers with a reference point for the level of input DNA quality required to obtain similar assembly outcomes, which is particularly helpful for those working on non-model or difficult-to-extract species.

 Beyond this, I do not have any major concerns regarding the manuscript. The authors have done a commendable job in assembling and describing this genome, and I support its publication after the minor suggestion above is addressed.

Are sufficient details of methods and materials provided to allow replication by others?

Yes

Is the rationale for creating the dataset(s) clearly described?

Yes

Are the datasets clearly presented in a useable and accessible format?

Yes

Are the protocols appropriate and is the work technically sound?

Yes

Reviewer Expertise:

Genomics

I confirm that I have read this submission and believe that I have an appropriate level of expertise to confirm that it is of an acceptable scientific standard.

# Reviewer report: Reviewer response for version 1

**DOI:** 10.21956/wellcomeopenres.26752.r124883

**Published:** 2025-06-23

**Authors:** Panagiotis Ioannidis

**Affiliations:** 1 Foundation for Research & Technology - Hellas, Crete, Greece

This paper by Sivell et al describes the sequencing and assembly of the genome of the soldierfly Chloromyia formosa. All quality metrics demonstrate that the genome assembly is of excellent quality. The only thing missing is an RNAseq library which would help in gene prediction.

 Minor comments:

 "...sequencing data provided approximately 34 coverage" should be "...sequencing data provided approximately 34x coverage"

 In "Assembly statistics" the authors mention: "These interventions decreased the scaffold count by 16.79% and increased the scaffold N50 by 98.92%". I think that mentioning the absolute numbers instead of the percentages is much more useful for the reader.

Are sufficient details of methods and materials provided to allow replication by others?

Yes

Is the rationale for creating the dataset(s) clearly described?

Yes

Are the datasets clearly presented in a useable and accessible format?

Yes

Are the protocols appropriate and is the work technically sound?

Yes

Reviewer Expertise:

insects genomics, bioinformatics

I confirm that I have read this submission and believe that I have an appropriate level of expertise to confirm that it is of an acceptable scientific standard.

# Reviewer report: Reviewer response for version 1

**DOI:** 10.21956/wellcomeopenres.26752.r124877

**Published:** 2025-06-14

**Authors:** Komal Kumar Raja

**Affiliations:** 1 Baylor College of Medicine, Houston, Texas, USA

The article presents the genome sequence of the Broad Centurion soldierfly, *Chloromyia* *formosa* with a total length of 790.81 megabases. The authors briefly present the morphological and ecological features of this species in the introduction. The assembly quality metrics are great with 95% BUSCO and k-mer completeness of 99.1%. The Hi-C contact map looks quite good with clean separation of distinct blocks as expected in Hi-C maps of well-separated chromsomes. Overall, the data is a good genomic resource.

Are sufficient details of methods and materials provided to allow replication by others?

Yes

Is the rationale for creating the dataset(s) clearly described?

Yes

Are the datasets clearly presented in a useable and accessible format?

Yes

Are the protocols appropriate and is the work technically sound?

Yes

Reviewer Expertise:

Single cell genomics, bioinformatics, genomics and Neurobiology

I confirm that I have read this submission and believe that I have an appropriate level of expertise to confirm that it is of an acceptable scientific standard.

# Reviewer report: Reviewer response for version 1

**DOI:** 10.21956/wellcomeopenres.26752.r124881

**Published:** 2025-06-05

**Authors:** Kyle M Benowitz

**Affiliations:** 1 Arizona State University, Tempe, Arizona, USA

The manuscript presents a chromosome-level genome assembly of a dipteran species using standard methods of HiFi and Hi-C sequencing. The assembly is clearly of excellent quality, and I have no issues with the description of the methods or the presentation of the assembly. It's worth noting that there is no annotation provided; this could limit the usefulness of the genome for certain comparative analyses.

 I do wish there was a bit more rationale provided for the sequencing of this species. The only justification given is that, as a species native to Britain, it falls under the remit of the DToL project to sequence all species in the British islands. This is fine, but nonetheless, I am curious to know whether there are any biological or phylogenetic reasons why having a genome for this species is important. What specific questions can this resource be helpful in addressing?

Are sufficient details of methods and materials provided to allow replication by others?

Yes

Is the rationale for creating the dataset(s) clearly described?

Partly

Are the datasets clearly presented in a useable and accessible format?

Yes

Are the protocols appropriate and is the work technically sound?

Yes

Reviewer Expertise:

Insect genomics; behavior; evolution

I confirm that I have read this submission and believe that I have an appropriate level of expertise to confirm that it is of an acceptable scientific standard.
